# An immune-competent human gut microphysiological system enables inflammation-modulation by *Faecalibacterium prausnitzii*

**DOI:** 10.1038/s41522-024-00501-z

**Published:** 2024-03-29

**Authors:** Jianbo Zhang, Yu-Ja Huang, Martin Trapecar, Charles Wright, Kirsten Schneider, John Kemmitt, Victor Hernandez-Gordillo, Jun Young Yoon, Mathilde Poyet, Eric J. Alm, David T. Breault, David L. Trumper, Linda G. Griffith

**Affiliations:** 1 Department of Biological Engineering, Massachusetts Institute of Technology, Cambridge, MA USA; 2 Swammerdam Institute for Life Sciences, University of Amsterdam, Amsterdam, The Netherlands; 3 Tytgat Institute for Liver and Intestinal Research, Amsterdam Gastroenterology, Endocrinology and Metabolism, Amsterdam UMC, Location Academic Medical Center, Amsterdam, the Netherlands; 4 Department of Mechanical Engineering, Yonsei University, Seoul, South Korea; 5 Department of Pediatrics, Harvard Medical School, Boston, MA USA; 6 Department of Mechanical Engineering, Massachusetts Institute of Technology, Cambridge, MA USA; 7 Center for Gynepathology Research, Massachusetts Institute of Technology, Cambridge, MA USA; 8 Present Address: Institute of Experimental Medicine, University of Kiel, Kiel, Germany

**Keywords:** Microbiota, Bacteria

## Abstract

Crosstalk of microbes with human gut epithelia and immune cells is crucial for gut health. However, there is no existing system for a long-term co-culture of human innate immune cells with epithelium and oxygen-intolerant commensal microbes, hindering the understanding of microbe-immune interactions in a controlled manner. Here, we established a gut epithelium-microbe-immune (GuMI) microphysiological system to maintain the long-term continuous co-culture of *Faecalibacterium prausnitzii/Faecalibacterium duncaniae* with colonic epithelium, antigen-presenting cells (APCs, herein dendritic cells and macrophages), and CD4^+^ naive T cells circulating underneath the colonic epithelium. In GuMI-APC condition, multiplex cytokine assays suggested that APCs contribute to the elevated level of cytokines and chemokines secreted into both apical and basolateral compartments compared to GuMI condition that lacks APC. In GuMI-APC with *F. prausnitzii* (GuMI-APC-FP), *F. prausnitzii* increased the transcription of pro-inflammatory genes such as toll-like receptor 1 (*TLR1*) and interferon alpha 1 (*IFNA1*) in the colonic epithelium, without a significant effect on cytokine secretion, compared to the GuMI-APC without bacteria (GuMI-APC-NB). In contrast, in the presence of CD4^+^ naive T cells (GuMI-APCT-FP), *TLR1*, *IFNA1*, and *IDO1* transcription levels decreased with a simultaneous increase in *F. prausnitzii*-induced secretion of pro-inflammatory cytokines (e.g., IL8) compared to GuMI-APC-FP that lacks T cells. These results highlight the contribution of individual innate immune cells in regulating the immune response triggered by the gut commensal *F. prausnitzii*. The integration of defined populations of immune cells in the gut microphysiological system demonstrated the usefulness of GuMI physiomimetic platform to study microbe-epithelial-immune interactions in healthy and disease conditions.

## Introduction

The human colonic mucosal barrier is a microarchitecture that acts as a physical barrier to harmful pathogens and as a coordinator of homeostatic crosstalk between microbiota and immune cells^1^. Compared to the small intestinal epithelium in which the villi structure can protrude up to 1 mm^2^, the colonic epithelial surface is relatively flat and composed of a single layer of cells, interspaced with invaginations called crypts where stem cells reside^3,4^. This colonic epithelial monolayer consists of several cell types, including colonocytes, goblet cells, Tuft cells, and enteroendocrine cells^1^. These cells communicate with luminal microbiota by actively metabolizing microbial metabolites (e.g., butyrate) and secreting host molecules (e.g., mucin). Likewise, epithelial cells communicate with innate immune cells which leads to the release of cytokines/chemokines or the secretion of IgA to prevent the body from bacterial invasion^5,6^. Antigen-presenting cells (APCs), including dendritic cells and macrophages, are essential for immune tolerance and protective immunity in the intestine. These APCs are differentially modulated by the microbiota to perform distinct functions such as, communicating with T cells and releasing context-specific cytokines^7^. Disruption of this microbiota-epithelium-immune axis can lead to inappropriate immune responses, which is believed to contribute to the development or progression of inflammatory diseases, including inflammatory bowel diseases^8^. However, the precise role of each component in the microbiota-epithelium-immune axis remains elusive, mainly owing to the lack of an appropriate model to disentangle the complex interactions.

While there is progress being made in developing human in vitro microfluidic gut models to mimic the microbiota-immune interplay, current in vitro microfluidic systems often use cancerous cell lines^9^, lack immune components^10^ or use PBMCs^11^ comprising an undefined mixture of immune cells. In addition, these microfluidic gut chips are fabricated using polydimethylsiloxane (PDMS)^10,11^, a material that is highly adsorptive of lipophilic small molecules or proteins^12–14^ that are critical for the innate immune responses.

The discrepant oxygen requirement for bacteria and epithelium further expands these challenges: the majority of >1000 bacterial species in the colon are intolerant to oxygen, whereas human intestinal and immune cells require oxygen. We recently established a primary human cell-derived PDMS-free gut-liver physiomimetic system and demonstrated a reliable co-culture of differentiated colonic epithelium, APCs, and regulatory T cells for studying inflammatory bowel disease^15^ and neurodegenerative diseases^16^. A new gut epithelium-microbe-immune (GuMI) microphysiological system was developed in parallel^17^. GuMI enabled the continuous culture of colonic epithelium with the extremely oxygen-sensitive bacterium *Faecalibacterium prausnitzii*, which is one of the most abundant bacterial species in human adult colonic microbiota^18^ and has important implication in reducing the risk of inflammatory diseases^19,20^.

Here, we describe the establishment of an immune-competent GuMI platform for co-culturing three types of immune cells (dendritic cells, macrophages, and CD4^+^ naive T cells) with a primary human colonic epithelium and *F. prausnitzii* over 48 hours. We examined the influence of human monocyte-derived APCs, i.e., dendritic cells and macrophages, on the phenotype of the colon mucosal barrier and bacterial growth compared to GuMI without immune cells (GuMI-APC-FP vs. GuMI-FP), assessing barrier function, and cytokine profile. We then studied the effects of *F. prausnitzii* in a multi-day interaction with primary human colonic epithelium, APCs, and CD4^+^ naive T cells (GuMI-APCT-FP). By measuring the gene transcription and cytokine secretion, we found the specific effects of *F. prausnitzii* on APC-mediated immune responses. In addition, including CD4+ naive T cells in the system reduces transcription of pro-inflammatory genes in the epithelium but increases cytokine secretion to the luminal side of the colonic epithelium. Collectively, this data suggest that GuMI model presented here can be used to dissect complex gut-immune-bacteria interactions.

## Results and discussion

### Antigen-presenting cells do not disrupt the integrity of colonic epithelium nor the growth of *F. prausnitzii*

Epithelial mucosal barriers regulate their homeostasis and response to microbiota in part by collaboration with innate immune cells and polarized production of growth factors, chemokines, and cytokines^21^. Most of these factors act not only in a paracrine fashion to recruit immune cells and signal neighboring stromal cells but also in an autocrine fashion: colonic epithelial cells and innate immune cells express not only canonical growth factor receptors (e.g., epidermal growth factor receptor, EGFR; fibroblast growth factor receptors, FGFRs; platelet-derived growth factor receptors, PDGFRs) but also receptors for chemokines (CXCR1-4; CCR2-5) and cytokines (IL1, IL4, IL15, and IL18)^22–30^. Autocrine loops regulate colonic epithelial barrier permeability, proliferation, response to infection, and diverse other behaviors, and in turn, the activity of autocrine loops is influenced by gut microbes^21^.

**Fig. 1:**
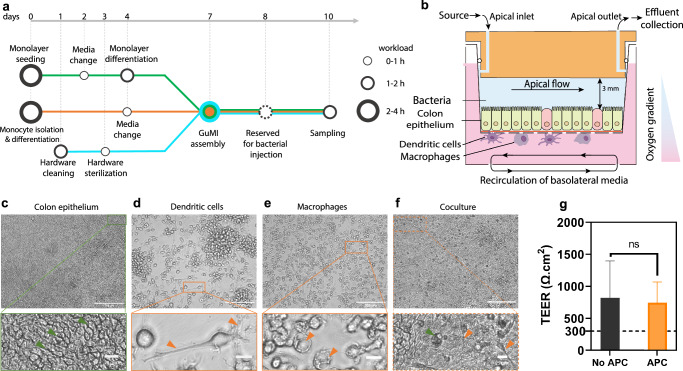
Establishment and characterization of the co-culture of colonic epithelium, dendritic cells, and macrophages in the GuMI system. **a** Workflow of colonic epithelial monolayer generation (green line), monocyte isolation, and differentiation to antigen-presenting cells (APCs, i.e., dendritic cells and macrophages; orange line), GuMI hardware preparation (aqua line), GuMI device assembly, operation, and sampling (merged lines). Circles in the metro map indicate the critical tasks and the workload. **b** Illustration of designed co-culture of primary colonic epithelium with APCs in GuMI (GuMI-APC). **c**–**f** Brightfield images of colonic epithelial monolayer, dendritic cells, macrophages, and co-culture. **c** Colonic epithelium without APCs in GuMI. Green arrows indicate the clear cell border among the epithelial cells. **d** Dendritic cells and **e** macrophages before adhering to the bottom of the semi-permeable membrane in the transwell insert with pore size of 0.4 μm. Orange arrows indicate the dendrite and phagosome-like structures in **d**, **e**. Green arrows in **c** indicates the border of epithelial cells. Green and yellow arrows in **f** indicates the shading of immune cells underneath the epithelium. Scale bars in **c**–**f**: 300 µm. **g** Transepithelial electrical resistance (TEER) values of the monolayer in GuMI-APC (orange bar) and GuMI (black bar) after 72 h in GuMI. The error bar indicates the standard deviation. *n* = 3. ns: not significant, two-tailed unpaired *t* test.

We previously established a GuMI system to co-culture colonic epithelium and anaerobic bacteria. However, the system lacked immune components, hampering the study of crosstalk among epithelial cells, immune cells, and bacteria. Reasoning that cytokine and chemokine secretion are essential markers and regulators of the immune responses to the gut microbiota and the lack of immune cells in the GuMI system leads to a negligible level of cytokines and chemokines (Supplementary Table 1), we integrated innate immune cells into the GuMI system. We isolated the monocytes from human primary peripheral blood mononuclear cells (PBMCs) and deliberately differentiated them into two types of antigen-presenting cells (APCs), namely dendritic cells and macrophages (more details in Methods, Fig. 1a). These APCs have been characterized in our previous studies^15,16^.

To build the GuMI, single colonic epithelial cells, derived from human colon organoids, were seeded on the apical side of a collagen-coated transwell and cultured for 4 days in expansion medium and 3 days in differentiation medium for a total of 7 days. In parallel, PBMCs were differentiated into dendritic cells and macrophages for 7 days to coincide with the colonic monolayer maturation. The dendritic cells and macrophages were then seeded on the basolateral side of the collagen-coated membrane that supports the colonic epithelial monolayers (Fig. 1a) before they were integrated into the GuMI platform. Once assembled, the GuMI platform created a physiological basal to apical oxygen gradient where the apical compartment was maintained anaerobic (Fig. 1b). Colonic epithelium and APCs were co-cultured in the sandwich-like co-culture to allow the epithelial-immune interaction (Fig. 1b). Brightfield microscopy examination confirmed the clear cell border of the differentiated colon monolayer after three days in GuMI (Fig. 1c). The morphological inspection confirmed the presence of dendritic cells and macrophages in GuMI-APC (Fig. 1d-f) but not in GuMI without APCs (Fig. 1c). These results suggest that dendritic cells and macrophages adhered to the bottom of the porous membrane and to the colonic epithelium. Notably, the transepithelial electrical resistance (TEER) values of epithelium with and without APCs were not significantly different after three days of co-culture (Fig. 1g), indicating that APCs do not alter the barrier function.

### Antigen-presenting cells are essential components of an immune-competent in vitro mesofluidic GuMI system

**Fig. 2:**
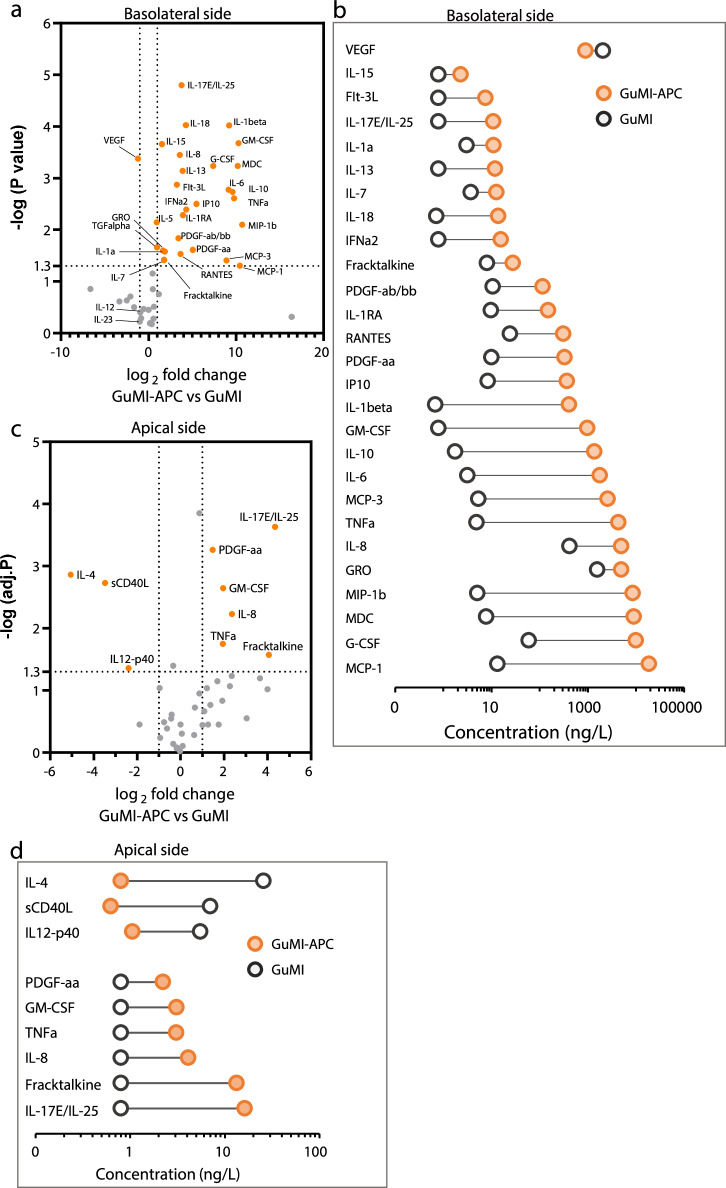
Antigen-presenting cells increase baseline cytokines in both apical and basolateral sides in the GuMI system. **a** Volcano plot analysis for the cytokine profile in the presence versus the absence of APCs (i.e., dendritic cells and macrophages) in the basolateral compartment of GuMI. Orange-filled circles indicate the significantly changed cytokines. Vertical dash lines marks the log_2_ fold change = 1, and horizontal dash lines marks the adjusted *p* value = 0.05. Multiple *t* test corrected using the Holm-Sidak method. **b** The list of significantly increased cytokines and decreased VEGF in the basolateral compartment of GuMI-APC (orange-filled circle) versus GuMI (black hollow circle). **c** Volcano plot of cytokine profile in the apical compartment of GuMI-APC versus GuMI. Orange-filled circles indicate the significantly changed cytokines. Vertical dash lines marks the log_2_ fold change = 1, and horizontal dash lines marks the adjusted *p* value = 0.05. Multiple *t* test corrected using the Holm-Sidak method. **d** The list of significantly increased and decreased cytokines in the apical compartment of GuMI-APC (orange-filled circle) versus GuMI (black hollow circle). *n* = 2, 3.

Next, we asked if the addition of immune cells will alter the baseline immune responses observed in the apical and basolateral sides of colon epithelial monolayers. We compared the secreted cytokine and chemokine profiles in the presence or absence of APCs in GuMI (Fig. 2). We determined the cytokine concentration in both apical and basolateral media collected 72 h after in GuMI-APC and GuMI conditions. In GuMI-APC, we used M-CSF human monocyte-derived macrophages that are classified as M2 macrophages, with characteristic production of IL10 and no production of IL12 and IL23^31^. It is worth noting that our GuMI platform is made of inert, gas-impermeable, polysulfone material that do not absorb hydrophobic compounds or proteins to overcome known limitations found in microfluidic devices made with PDMS^12,17,32^. Thus, we believe the cytokine and chemokines detected in our studies represent a better aproximation of the cell-secreted factors in the model.

The addition of APCs in the basolateral side resulted in significant changes in cytokine secretion. Of the 47 analyzed cytokines/chemokines, 28 were significantly increased (adjusted p < 0.05, │log2fold change│ ≥1), and one (VEGF) was significantly decreased in GuMI-APC versus GuMI (Fig. 2a). These analytes include hallmark cytokines secreted by dendritic cells and macrophages such as MCP1/CCL2 and IL10 (Fig. 2b). Notably, MCP1/CCL2 on the basolateral side increased from 13 ng/L to 18815 ng/L (Fig. 2b), suggesting APCs are the primary source of MCP1/CCL2. These results are consistent with previous observations that macrophages isolated from mouse intestinal lamina propria produce MCP1/CCL2 even without inflammation^33^, and intestinal epithelial cells also produce MCP1/CCL2^34^. Macrophages also play an essential role in intestinal homeostasis by producing anti-inflammatory cytokine IL10. Herein, we employed M-CSF-induced PBMC-derived macrophages, which is classified as IL10-producing M2 macrophages^31^. In GuMI-APC, a high amount (1385 ng/L) of IL10 but not IL12 or IL23 (Fig. 2a and b) was observed on the basolateral side. Similarly, Kamada et al. observed that M-CSF-induced bone marrow-derived macrophages and colonic lamina propria macrophages produced a high amount ( ~ 1500 ng/L) of IL10 but not IL12 or IL23 upon stimulation of heat-killed bacteria *Enterococcus faecalis*, whereas GM-CSF-induced counterparts secreted a large amount of IL12 and IL23^35^.

Dendritic cells also contribute to producing specific cytokines like IL6, IL18, TNFA, and GM-CSF/CSF2 (Figs. 2a and b). It was shown that dendritic cells can spontaneously express these genes, but not *IL2*, *IL3*, *IL4*, *IL5*, *IL9*, and *IFNG*^36^. Consistently, we did not observe significant increase of IL2, IL3, IL4, IL5, IL9, and IFNG proteins in the basolateral side of GuMI-APC. Importantly, both anti- and pro-inflammatory cytokines IL10, IL8, and TNFA were increased, likely due to the baseline activities of APCs. Both anti- and pro-inflammatory cytokines are maintained at a certain level in homeostasis in vivo in the colon^33^. This and the macrophage-derived cytokines suggest that macrophages and dendritic cells function under the oxygen gradient and fluidic microenvironment provided by our GuMI platform. It also demonstrates that APCs largely contribute to secreted growth factors, cytokines, and chemokines, highlighting the importance of APCs in establishing an immune-competent in vitro gut model.

In contrast to the basolateral side, we did not expect the detection of cytokines or chemokines in the apical side because the media was refreshed at a flow rate of 10 µl/min, equivalent to a ⁓123-fold dilution over two days. Surprisingly, integrating APCs in GuMI significantly increased the number and levels of detectable cytokines on the apical side (Fig. 2c), suggesting that APCs are metabolically active and have baseline immune responses. Interestingly, three cytokines, i.e., sCD40L, IL12-p40, and IL4 (Fig. 2d), were significantly decreased on the apical side of GuMI-APC. In the basolateral side, IL4 was slightly but not significantly (p = 0.07) higher in GuMI-APC vs. GuMI, while IL12-p40 and sCD40L remained at low levels in both GuMI-APC and GuMI. IL4 effectively promotes the differentiation of dendritic cells and is known to be consumed during the activation of dendritic cells^37^. Following IL4-mediated differentiation, dendritic cells can be driven to a more mature state by TNFA^38^. Consistently, TNFA is significantly increased on the apical side (Fig. 2c). Previous studies have reported that sCD40L, IL12-p40, and IL4 are essential for differentiating monocytes toward dendritic cells and macrophages. Together with our data, these results suggest that dendritic cells and macrophages consumed sCD4L, IL12-p40, and IL4. Notably, the APCs in the GuMI system apppears to secrete distinctive cytokines in response to other cytokines present in the system. For instance, six cytokines were significantly increased (Fig. 2d), including PDGF-AA, GM-CSF/CSF2, TNF, IL8/CXCL8, fractalkine/CX3CL1, and IL17E/IL25. These cytokines are characteristic markers of functioning APCs^39,40^. Other cytokines detected in our GuMI-APC model have been reported previously (Fig. 2). For instance, MIP-1A/CCL3 and MIP-1B belong to the MIP1 CC chemokine subfamily and were shown to be mainly secreted by dendritic cells and macrophages^41^. TNFA can increase secreted IL8/CXCL8 in the basolateral side^42^. IL8/CXCL8 is secreted and is an essential mediator of innate immune responses. In mice, colonic lamina propia macrophages produce a large amount of IL10 and MCP1/CCL2 in a steady state and an even higher level of MCP1/CCL2 in the inflammation site^33^.

### *F. prausnitzii* induces transcriptional immune responses in the colonic epithelium in the presence of APC

**Fig. 3:**
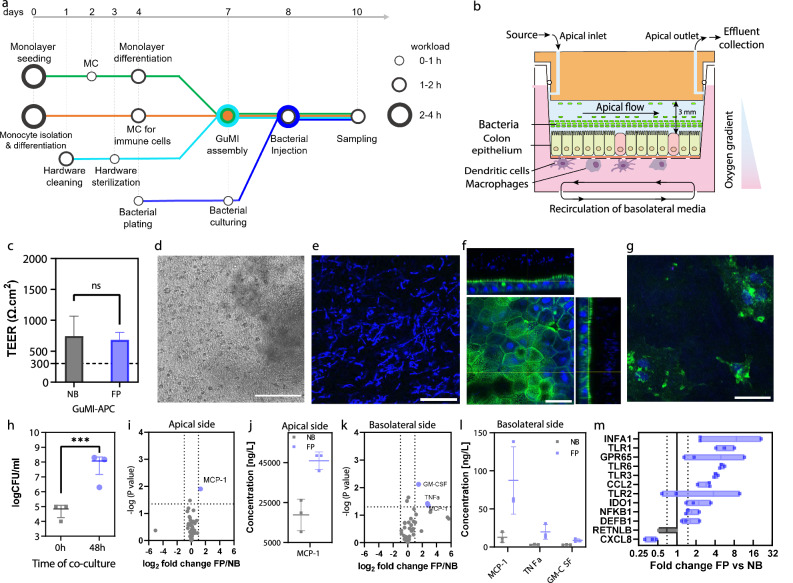
Cytokine and transcriptional changes induced by bacterium *F. prausnitzii* in GuMI with APCs. **a** Workflow of GuMI experiments including preparation of monolayer (green line), monocyte isolation and APC differentiation (orange line), hardware preparation (aqua), and bacterial culturing (blue line). Circles in the metro map indicate the critical tasks and the workload. Color blue in **c** and **h**–**m** is used to highlight the conditions with *F. prausnitzii*. **b** Schematic demonstration of co-culture of *F. prausnitzii*, colonic epithelium, and APCs. **c** TEER values of GuMI-APC with and without *F. prausnitzii*. Two-tailed unpaired *t* test. **d**–**g** Brightfield image (**d**), immunofluorescent staining of bacterial cells (**e**), epithelium (**f**), and APCs (**g**). Scale bar, 300 μm (**d**) and 30 μm (**e**–**g**). Staining, nucleic acids (blue, **e**–**g**) and green (beta-actin, **f**, **g**). **h** Live bacterial density of *F. prausnitzii* at 0 and 48 hours. ***: *p* < 0.001, two-tailed unpaired *t* test. **i** Volcano plot comparing cytokines/chemokines in apical media in the presence or absence of *F. prausnitzii* (GuMI-APC-FP vs. GuMI-APC-NB). **j** The significantly increased cytokine concentration in apical media shown in **i**. **k** Volcano plot on the comparison of cytokines or growth factors in basolateral media in the presence or absence of *F. prausnitzii* (GuMI-APC-FP vs. GuMI-APC-NB). **l** The concentration of significantly increased cytokine MCP1/CCL2 in basolateral media shown in **k**. **i**–**l** Multiple *t* test corrected by the Holm-Sidak method. **m** Transcriptional change of inflammation-related genes in colonic epithelial cells in GuMI-APC *F. prausnitzii* versus no bacteria. *n* = 3. Blue boxes indicate a <0.75-fold decrease or >1.5-fold increase, and black boxes indicate no significant difference. Two-tailed unpaired *t* test. Error bars in **c**, **h**, **j**, **l** represent standard deviation.

To further increase the complexity of our GuMI-APC model and recapitulate some aspect of the gut-microbiome-immune axis, we introduced *Faecalibacterium prausnitzii* bacteria into the apical compartment (Fig. 3a-b). *F. prausnitzii* is an oxygen-intolerant bacterial species found in the human adult gut microbiota^43^. Thus, we speculated that if the basolateral-apical oxygen gradient in the GuMI platform is maintained, it will allow the proliferation of *F. prausnitzii*.

The co-culture of *F. prausnitzii* in the GuMI-APC model required careful coordination and synchronization of different tasks (Fig. 3a, b). For instance, colon epithelial seeding and differentiation needs to happen in parallel with macrophage and dendritic cell differentiation so that immune cells can be seeded in the basolateral side by day 7 (Fig. 3a). Bacteria is introduced into the apical side on day 8 (16–18 hours after seeding the immune cells), but bacterial culture off-platform starts on day 4 (Fig. 3a)

To unambiguously determine if the apical side of the GuMI platform is permissive of oxygen-intolerant bacterial growth, we quantified the fold increase in live bacteria using the colony-forming unit protocol described in the Methods section. Since we speculated that an intact monolayer is needed to prevent oxygen from leaking from the basolateral to the apical side, we assessed epithelial barrier function using TEER measurement and visual inspection under a microscope. No significant change was observed in the TEER values (Fig. 3c), and no observable holes were observed under microscopic examination (Fig. 3d–g), confirming that the monolayers were intact with or without *F. prausnitzii*. The intact epithelial barrier allowed the growth of *F. prausnitzii* during the 48 hours of co-culture in GuMI-APC. Colony plating indicated a more than 1000-fold increase in the density of bacteria in the apical compartment of GuMI-APC, which reached ~10^8^ CFU/ml (Fig. 3h). This result indicates an active bacterial growth in GuMI-APC. The final bacterial density is similar to that in the absence of APCs^17^, suggesting that the introduction of APCs does not influence the growth of *F. prausnitzii*. These results suggest that GuMI can maintain the co-culture of bacteria-epithelium-APCs leading to an immune-competent GuMI platform.

To further investigate the spatial organization of the different cells in GuMI-APC, we performed nucleic acid (blue) and actin (green) staining, followed by confocal imaging (Fig. 3e–g). Confocal imaging revealed that bacterial cells (blue staining Fig. 3e**)** were on top of the colonic epithelium. Further, orthogonal projection (Fig. 3f) demonstrated a bacterial cell “layer” and beneath, an epithelial monolayer. The monolayer displayed typical cobblestone arrangement, indicative of epithelial cells (Fig. 3f), similar to the colonic epithelium in other reports^15,17,44,45^. Imaging of the basolateral side showed cells with irregular shapes and tentacle-like extensions (Fig. 3g) which are typical morphology of APCs, such as dendritic cells.

Next, we sought to investigate the impact of *F. prausnitzii* on the immune responses of colonic epithelium in the presence of APCs. We compared the concentration of 47 cytokines in the apical and basolateral compartments of GuMI-APC-FP vs. GuMI-APC-NB (no bacteria, Fig. 3). Surprisingly, most cytokines remained similar to the baseline levels in GuMI-APC-NB (Fig. 3i and j). Only three cytokines were significantly increased in the apical and one in the basolateral compartments of GuMI-APC-FP, respectively (Fig. 3i and k). The levels of MCP1/CCL2 in both apical and basolateral media were significantly increased by *F. prausnitzii* (Fig. 3j and l). Others have shown that *Lactobacilli* and *Streptococci* bacteria also induce MCP1/CCL2 production in human macrophages^46^. Moreover, other reports suggest that MCP1/CCL2 is critical in recruiting monocytes to the inflammation site^47^. MCP1/CCL2 protein is constitutively secreted in the normal intestinal colonic mucosa and is upregulated in patients with ulcerative colitis or Crohn’s disease^34^. Consistently, the mRNA level of *MCP1/CCL2*, 2.9-fold) was significantly increased in colonic epithelial cells in GuMI-APC-FP (Fig. 3m). This result agrees with the clinical observations, where *MCP1/CCL2* mRNA levels were markedly increased in inflamed intestinal biopsies from patients with inflammatory bowel disease^34^. In addition to MCP1/CCL2, TNFA protein was also increased in GuMI-APC-FP (Fig. 3l). Recently, it was found that 10% of *F. prausnitzii* fermented supernatant increased the protein level of TNFA in LPS-pretreated colonic HT29 cells^48^. These results indicate that *F. prausnitzii* homeostatically activates immune and epithelial cells in the GuMI-APC, with increased secretion of a few pro-inflammatory cytokines. To test this hypothesis, we looked at the expression of genes that are critical for inflammation regulation such as *TLR*s, *IDO1*, *IFNA1*, *CXCL8/IL8*, and *NFKB1* in colonic epithelium. TLRs mediate host cell recognition of virus, pathogens, and commensal bacteria^49^, with genes such as *IDO1* and *IFNA1* regulating *TLR* gene expression^50,51^. Transcription of *IDO1* is significantly higher in the ileum and colon in models of inflammation induced by immunostimulatory DNA (CpG), TNBS, and DSS^52^. Mice deficient in *IDO1* repress the activation of the *TLR-Myd88-NFKB1* network and thus developed less severe colitis induced by DSS^51^. Herein, we found that the transcription of *TLR1*, *TLR3*, *TLR6*, and *NFKB1* was significantly upregulated in colonic epithelial cells upon exposure to *F. prausnitzii* (Fig. 3m). Consistently, the transcription of *IDO1* and *IFNA1* was increased by *F. prausnitzii*. The activation of *IDO1* in colonic epithelial cells agrees with previous observations on *F. prausnitzii*-mediated activation of dendritic cells^53^. When dendritic cells were exposed to a single dose of dead *F. prausnitzii* cells, *IDO1* and TLRs (i.e., *TLR2* and *TLR4*) were activated at the transcriptional level^53^. Despite the fact that *F. prausnitzii* unexpectedly decreased the mRNA level of *CXCL8/IL8* (0.4-fold, Fig. 3m) with no change in CXCL8/IL8 secretion, these results indicate that in the presence of APCs, *F. prausnitzii* primes colonic epithelial cells to be transcriptionally activated toward bacteria-activating and pro-inflammatory states, but to secrete only a few pro-inflammatory cytokine proteins.

### CD4^+^ naive T cells increase the *F. prausnitzii*-induced secretion of cytokines and decrease the transcription of TLR in the colonic epithelium

**Fig. 4:**
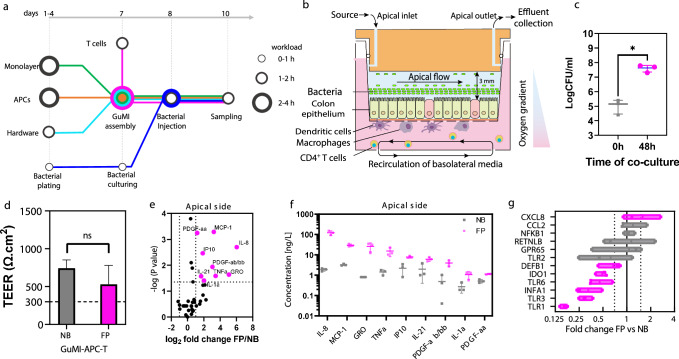
Integration of CD4^+^ naive T cells in GuMI-APC demonstrates the contribution of CD4^+^ naive T cells in the systemic immune response to bacterium *F. prausnitzii.* **a** Workflow to establish GuMI-APCT with and without *F. prausnitzii*. **b** Illustration of designed co-culture of colonic epithelium, APCs, CD4^+^ T cells, and *F. prausnitzii* in the GuMI platform. Magenta highlights the inclusion of CD4^+^ T cells. **c** Live bacterial density of *F. prausnitzii* at 0 and 48 hours. CFU colony-forming unit. *p < 0.05, two-tailed unpaired *t* test. Magenta in **c**–**g** indicates the effects of *F. prausnitzii* in the presence of CD4^+^ T cells. **d** The introduction of CD4^+^ T cells does not influence the TEER values of the monolayer in GuMI. TEER transepithelial electrical resistance. FP *F. prausnitzii*, NB no bacteria. ns not significant, two-tailed unpaired *t* test. **e** The volcano plot compares cytokines/chemokines in apical media in the presence or absence of *F. prausnitzii* (GuMI-APCT-FP vs. GuMI-APCT-NB). **f** Significantly increased cytokines in apical media induced by *F. prausnitzii* in GuMI-APCT. **e**, **f** Multiple *t* test corrected by the Holm–Sidak method. **g** Transcriptional change of selected inflammation-related genes induced by *F. prausnitzii* in GuMI-APCT. Magenta boxes indicate a <0.75-fold decrease or >1.5-fold increase, and black boxes indicate no significant difference. Two-tailed unpaired *t* test. Error bars in **c**, **d**, **f** represent standard deviation.

In the intestinal mucosal barrier, the interaction of APCs and T cells is crucial in the intestinal innate immunity in response to microbiota. A recent single-cell survey revealed that T cells (including CD4^+^ T cells, Th1 helper cells, Th17 cells, and other Treg subtypes) account for a considerable proportion of the immune cell population in human colon mucosa^54^. Reasoning that adding T cells will likely close the communication gap among the epithelium, APCs, and T cells responding to the gut microbiota, we integrated CD4^+^ naive T cells into the established GuMI-APC to generate GuMI-APCT co-culture and exposed the system to *F. prausnitzii* (GuMI-APCT-NB *vs*. GuMI-APCT-FP, Fig. 4a). The CD4^+^ naive T cells were introduced into the GuMI platform at day 7 after seeding epithelial cells (see timeline in Fig. 4a**)**. The pumping system allows the recirculation of CD4^+^ T cells in the basolateral compartment without causing cell death or damage to the cells present in the GuMI-APC model (Fig. 4b)^15,55^, and the designed co-culture enables the interactions of colonic epithelial cells, APCs, CD4^+^ T cells, and bacterial cells (Fig. 4b). As a quality control, we first examined if *F. prausnitzii* grows in GuMI-APC**T** to a similar extent as in GuMI-APC. At 48 h after bacterial introduction, the live bacterial density in the apical compartment reached ~10^8^ CFU/ml in GuMI-APC**T**-FP, which is similar to that in GuMI-APC-FP (Fig. 4c) and that of *F. prausnitzii* in human fecal samples (2.5 × 10^7^ – 7.9 × 10^11^ gene copies/g feces)^56^ and mouse intestine (3.4 × 10^8^ to 2 × 10^9^ CFU/g)^57^. Consistently, the integrity of the colonic epithelial monolayer was not affected by CD4^+^ naive T cells, evidenced by the similar TEER values above 300 Ω cm2 (Fig. 4d), an empirical threshold for an intact epithelial barrier in vitro^45,58^. These results confirmed that naive CD4^+^ T cells do not affect bacterial growth nor epithelial barrier integrity.

Next, we sought to investigate how the GuMI-APC**T** responds to *F. prausnitzii*. We first looked at the changes in cytokines induced by *F. prausnitzii*. Nine of 47 cytokines/chemokines in the apical compartment were significantly increased by *F. prausnitzii*, while no cytokines/chemokines were significantly decreased (Fig. 4e). TNFA and MCP1/CCL2 were increased by *F. prausnitzii* in GuMI-APCT, similar to that in GuMI-APC. With the presence of CD4^+^ naive T cells, six other cytokines, i.e., IL8, GRO, IP10, IL21, PDGF-AB/BB, IL1A, and PDGF-AA (Fig. 4f), were increased in response to *F. prausnitzii*. The cytokines induced by *F. prausnitzii* are typically regarded as pro-inflammatory cytokines. However, the levels of these cytokines are 5- to 20-fold lower to be considered as hyper-inflammation (Fig. 4f), in which cytokines and chemokines level such as TNFA, IP10, and PDGF-BB in the plasma of cytokine storm syndrome patients are > 100 ng/L, > 1000 ng/L, and > 2000 ng/L, respectively^59^. In fact, commensal gut microbiota has been shown to contribute to the training of our immune system by inducing baseline inflammation during homeostasis^60^. Nevertheless, including the CD4^+^ T cells enhances the cytokine-mediated immune responses to *F. prausnitzii*, suggesting active communication among APCs, T cells, and epithelial cells. Transcriptional changes in epithelial cells further support this notion. In the presence of naive CD4^+^ T cells, *F. prausnitzii* downregulated the transcription of *IDO1* (0.49-fold) and *IFNA1* (0.33-fold) in colonic epithelial cells (Fig. 4g). Similarly, TLRs, the downstream genes of *IDO1* and *IFNA1*, were decreased: *TLR1* (0.16-fold), *TLR2* (0.63-fold), *TLR3* (0.30-fold), and *TLR6* (0.45-fold) (Fig. 4g). Importantly, *NFKB1* expression was not affected (1.1-fold). These results suggest that *F. prausnitzii* is lowering the inflammation state at the transcriptional level in the presence of naive CD4^+^ T cells. Compared to the circumstance when only APCs were present, the addition of naive CD4^+^ T cells reverses the *F. prausnitzii*-mediated effects in colonic epithelium at the transcriptional level, highlighting the importance of T cells in coordinating the innate immune response to *F. prausnitzii* in the GuMI system. Interestingly, *IDO1* is significantly higher in ileal tissue from Crohn’s Disease patients with active inflammation but not without active inflammation^52^. Transcription of *IDO1* is significantly higher in the ileum and colon in rodent models of inflammation induced by immunostimulatory DNA (CpG), TNBS, and DSS^52^. It has been shown previously that *TLR3* and *TLR4* were significantly downregulated by *F. prausnitzii*-produced butyrate during its co-culture with colonic epithelium in the same GuMI physiomimetic platform^45^. *F. prausnitzii* can produce several types of anti-inflammatory molecules such as butyrate, MAM^61^, and sialic acid^57^. In animal models, *F. prausnitzii* alleviated the IBD symptoms^62^. Our results agree with these observations and demonstrate the contribution of naive CD4^+^ T cells in the interaction of the epithelium, innate immune components, and *F. prausnitzii*.

In summary, we established a microphysiological system that enables the co-culture of primary human colonic epithelium with monocyte-differentiated antigen-presenting cells (dendritic cells and macrophages), CD4^+^ naive T cells, and oxygen-sensitive gut commensal *F. prausnitzii*. Using multiplex cytokine assays and RT-qPCR, we demonstrated that the antigen-presenting cells substantially contribute to maintaining systemic immune cytokines. On top of that, circulating CD4^+^ naive T cells alter cytokine-mediated immune responses to *F. prausnitzii* and transcription of microbe-recognition genes. The results demonstrate the successful integration of three types of immune cells that are important in the innate immune-epithelium-microbiota axis and reveal the contribution of individual types of immune cells in response to gut commensal *F. prausnitzii*. These findings elucidate the critical role of CD4^+^ T cells that may maintain tolerance to intestinal microbiota by rendering the sensitivity of APCs and intestinal epithelial cells to commensal bacteria through the downregulation of pro-inflammatory genes. Finally, the established system provides a new tool to study microbe-host-immune interactions.

### Limitations of the study

While three types of immune cells are included in GuMI, other cell types such as fibroblasts, mast cells, and sensory neurons, have implications for gastrointestinal disorders^63,64^. The bacterial species *F. prausnitzii*, together with *B. thetaiotaomicron* and *Eubacterium rectale*^17^, are representatives of adult gut microbiota, and further study is warranted to include complex microbiota such as synthetic microbial communities^65^. Our pilot experiment demonstrated that it is feasible to co-culture synthetic microbial communities with colonic epithelium in GuMI (Supplementary Methods, Supplementary Note, and Supplementary Figure 1). In addition, fungi and bacteriophages are also essential components of gut microbiota^66–68^. The inclusion of them in the GuMI system will offer a new avenue for studying multi-kingdom interactions in gut health. Finally, including stem cell niche^69^ in the system may offer an even more comprehensive capture of the architecture of the intestine.

## Methods

### Bacterial culture

*Faecalibacterium prausnitzii* A2-165 (also known as *Faecalibacterium duncaniae* DSM17677) was obtained from the Harvard Digestive Disease Center. The strain’s identity was confirmed using Sanger sequencing (see below). Bacteria from glycerol stock were plated in yeast casitone fatty acid (YCFA) agar containing carbohydrates (Anaerobe Systems, AS-675) for 24-48 h after being cultured at 37 °C in the incubator inside the anaerobic chamber (Coy Laboratory), and a colony was picked and cultured in Hungate tubes containing liquid YCFA medium containing carbohydrates (Anaerobe Systems, AS-680). O_2_ in the anaerobic chamber was constantly removed by the Palladium Catalyst (Coy Laboratory, 6501050), which was renewed biweekly by incubating the catalyzer in the 90 °C oven for two days.

### PCR and Sanger sequencing

Bacteria identity was confirmed by Sanger sequencing by following the established protocol^45^. Briefly, bacterial cells were pelleted by centrifugation (12,000 *g* × 5 min). The DNA was extracted using the GeneElute bacterial DNA kit (NA2110, Sigma-Aldrich) following the manufacturer’s protocol. Afterward, PCR was performed in triplicate to amplify 16 s rDNA using DreamTaq Green PCR Master Mix (K1081, Thermo Fisher Scientific Inc.) with primers F8 (5’-AGTTTGATCCTGGCTCAG-3’) and 1492 R (5’-TACGGYTACCTTGT TACGACTT-3’) by following the procedures described elsewhere^70^. PCR products were sent for Sanger sequencing after DNA purification (Genewiz Inc.). The identity of the bacteria was confirmed to be *F. prausnitzii* DSM17677 using Blastp (Supplementary Figure 2).

### Colonic epithelial monolayer

#### Colon organoid and monolayer culture

Primary human colon organoids and monolayers were established and cultured according to previously described protocols^45,71^. The organoids were derived from endoscopic tissue biopsies taken from a patient (the normal appearing region of rectosigmoid sample from a 30-year-old male patient for diverticulosis and diverticulitis) upon informed consent. Methods performed followed the Koch Institute Institutional Review Board Committee and the Massachusetts Institute of Technology Committee on using humans as experimental subjects. The medium for maintaining organoids and monolayers includes a base medium, organoid growth medium, seeding medium, and differentiation medium. The recipe for each medium and the reagents are listed in Supplementary Tables 2 and 3. In brief, organoids in Matrigel (growth factor reduced, phenol red-free; Corning, 356231) droplets were grown in 24-well tissue culture-treated plates (Olympus Plastics, 25-107) and passaged every seven days at a 1:3 split ratio. A medium change was performed on day four using organoid growth medium after passaging. To prepare the monolayer, organoids were collected on day 7 and pelleted by centrifugation (1000 *g* × 5 min, 4°C), followed by Matrigel digestion using Cell Recovery Solution (Corning, 354253; 1 mL per 100 µL Matrigel). The resulting organoid suspension was then incubated on ice for 45-60 min, pelleted, and then digested at 37 °C for 5 min using 1 mL Trypsin/EDTA (2.5 mg/mL Trypsin [Sigma, T4549] and 0.45 mM EDTA [Ambion, AM9260G] in PBS without calcium and magnesium [PBS^−/−^, Gibco, 10010-023]). The digested organoids were manually dissociated into single cells using a 1000-µL pipette with a bent tip. The resulting cell suspension was then pelleted (300 *g* × 5 min, 4 °C) after neutralizing Trypsin was neutralized with 10% FBS in the base medium. The cell pellet was resuspended in the seeding medium and seeded in collagen I-coated (Gibco, A10483-01, 50 µg/mL in PBS) 12-well Transwells (Corning 3460, 0.4 µm pore polyester membrane). The seeding cell density was 300,000 cells per well (surface area: 1.12 cm^2^). Around 72 hours after seeding, the monolayers were differentiated by switching to the antibiotic-free base medium on the apical side and the differentiation medium on the basolateral side. After switching to a differentiation medium, the monolayers were cultured for four days (a total of 7 days), with medium change on day 5. The monolayers were used for experiments on day seven after seeding (day four after differentiation).

### Generation of dendritic cells, macrophages, and CD4^+^ T cells

#### Isolation and differentiation of monocytes

Monocytes were isolated from PBMC. PBMC were isolated from fresh whole blood with CPDA-1 anticoagulant (Research Blood Components LLC) using the SepMate PBMC Isolation Kit (StemCell, 85450) following the manufacturer protocol. After isolation, the PBMC were suspended in an immune cell freezing medium (RMPI with 10% dimethyl sulfoxide [DMSO] and 10% heat-inactivated FBS) and frozen at −196 °C.

Monocytes were isolated and differentiated into dendritic cells and macrophages on the 7th day before device assembly (see details in Device assembly and operation) for 7 days^16^. First, the PBMC were thawed in a 37 °C water bath for ~1 minute before diluting 1:10 in PBS^−/−^ containing 2% heat-inactivated FBS (PBS-HIFBS). After that, cells were pelleted (300 *g* × 5 min, 4 °C) and then resuspended in 1 mL of PBS-HIFBS, followed by a transfer into a 5-ml round-bottom polystyrene tube (StemCell, 100-0088). An additional 1.5 mL PBS-HIFBS was used to recover the residual cells and transferred into the same polystyrene tube. The isolation of monocytes was performed using the EasySep™ Human Monocyte Enrichment Kit without CD16 depletion (StemCell, 19058) and EasySep™ Magnet (StemCell, 18000). The resulting monocytes were split into two aliquots and pelleted (300 *g* × 5 min, 4 °C). The cell pellets were resuspended in the macrophage or dendritic cell differentiation media and cultured in 24-well tissue-treated plates. Both dendritic cells and macrophages were plated at a density of 1 × 10^6^ cells per well and in a volume of 500 μl per well. Four days after isolation and plating, 500 μl of MDM and DCDM were added to each macrophage and dendritic cell well, respectively. Dendritic cells were mixed gently via repeated pipetting upon media change to disrupt cell clumps, while macrophages were not mixed. 25,000 of each cell type were used to attach the membrane.

#### Isolation of CD4^+^ naive T cells

The CD4^+^ naive T cells were isolated from PBMC on the same day of device assembly (see details in Device assembly and operation). In brief, PBMC was thawed in a 37 °C water bath for ~1 minute before diluting 1:10 in PBS-HIFBS. After that dilution in the isolation buffer, centrifugation at 300 × *g* for 5 min and 4 °C was performed, and the isolation buffer was removed from the cell pellet. The cell pellet was then resuspended in 1 mL of PBS-HIFBS and transferred into a 5-ml round-bottom polystyrene tube. An additional 1.5 mL PBS-HIFBS was used to recover the residual cells and transferred into the same polystyrene tube. naive CD4^+^ T cells were isolated using the EasySep™ Human naive CD4^+^ T Cell Isolation Kit II (StemCell, 17555) and EasySep™ Magnet. Once isolated, the naive CD4^+^ T cells were pelleted and resuspended in 1 mL of RPMI 1640 supplemented with 10% HIFBS (RPMI-HIFBS) and ready for use. Cells were counted via Trypan Blue and Countess II Automated Cell Counter, and 60,000 naive CD4^+^ T cells were used in each well in circulation.

#### Co-culture of epithelial monolayers with dendritic cells, macrophages, and naive CD4^+^ T cells

In the experiments with APCs, i.e., dendritic cells and macrophages were harvested and seeded onto the basolateral side of the transwell membrane. To harvest the cells, cells were resuspended in their own media and collected into a conical tube (one tube per cell type). The residual cells were detached by adding 250 μl TrypLE Express (Gibco, 1260413) to each well and incubated at 37 °C for ~15 minutes, or until cells were detached from the plate. The TrypLE was then neutralized with 750 μl RPMI-HIFBS. The resulting cell suspensions were collected into the corresponding conical tubes. After that, the cells were pelleted (300 *g* × 5 min, 4 °C), resuspended in 1 mL of RPMI-HIFBS, and counted using trypan blue and countess. Dendritic cells and macrophages were then combined to achieve a density of 1.67 × 10^5^ cells per mL for each cell type. Before adding dendritic cells and macrophages, the media of the transwells was removed from both apical and basolateral sides, and each side was rinsed once with an antibiotic-free base medium. The transwells were then inverted and placed in a petri dish before adding 150 μl of the dendritic cell and macrophage cell suspension to each well to achieve a density of 0.25 × 10^5^ cells per transwell for each cell type. The transwells were then incubated at 37 °C for 2 hours to allow the attachment of dendritic cells and macrophages before proceeding with the further experimental setup.

In the experiments with naive CD4^+^ T cells, the freshly isolated naive CD4^+^ T cells were pelleted and resuspended using colon differentiation medium to achieve a density of 40,000 cells/mL (60,000 cells per well). The T cell-containing medium was added into the basolateral compartment in the GuMI device, where the naive CD4^+^ T cells are circulated.

### Device assembly and operation

The device assembly, operation, and sampling followed the previously described protocol^17^ with slight adaption in the experiments with immune cells. In brief, all components of the GuMI device were sterilized by autoclave (121 °C, 45 min), except the pneumatic plates, which were sterilized with ethylene oxide (Anprolene® AN74i, AN75.65,– Anderson Sterilizers Inc). Then, the device was assembled under sterile conditions. The GuMI apical medium (110 mL 10% YFCA in PBS^+/+^) was added to the apical source reservoir on top of the GuMI device (total capacity 150 mL). The medium in the apical source reservoir was then deoxygenized with 5% CO_2_ and 95% N_2_ for 45-60 min before being introduced into the apical inlet. After that, the apical inlet of the Transwell was temporally blocked with a 200-µl pipette tip to force the deoxygenized apical medium to flow out of the injection port, which was then sealed with an injection septum and a customized stainless-steel hollow screw. The pipette tips were then removed. The colonic epithelial monolayers were transferred to the six basolateral reservoirs prefilled with PBS^+/+^. The base medium in the apical side of the monolayers was replaced with the 10% diluted YCFA in PBS^+/+^. Then, the entire basal plate was integrated with the apical plate using the lever. In the experiment with APCs or APCs and naive CD4^+^ T cells, the inverted transwells were reversed and placed into basolateral reservoirs prefilled with PBS^+/+^. The basal plate was then disassembled using the lever, and the PBS was replaced with colon differentiation media, or CD4^+^ T cells colon differentiation media in the experiments with CD4^+^ T cells. The system was primed for 24 h in a cell culture incubator while the medium in the apical source reservoir was constantly purged with 5% CO_2_ and 95% N_2_. The recirculation flow rate in the basal compartment was 5 µl/min, and the apical flow rate was 10 µl/min. The effluent was cleared every 24 h with a 10-ml syringe (302995, BD Biosciences) throughout the experiments.

### Bacteria co-culture with colonic epithelial monolayers

The co-culture of bacterial cells with colonic epithelial monolayer was performed according to the established protocol^71^. Briefly, colonic epithelial monolayers were cultured in the GuMI device for 24 h before adding bacteria. In the experiments with APCs, the monolayers were replaced by the monolayers with dendritic cells and macrophages attached to the bottom of the porous polyester membrane. After that, the overnight grown bacterial cultures were diluted 1000 times with a pre-reduced anaerobic YCFA medium. Approximately 1 ml of the diluted bacterial cells was slowly injected into the apical channel using a 1-ml syringe (309659, BD Biosciences) with a needle (305127, BD Biosciences). After one hour of settling the bacterial cells, the flow resumed on both the apical and basolateral sides. After the experiment, the whole device was transferred to a biosafety cabinet, and the basal plate was carefully disassembled. The sealed Transwells were individually removed from the apical plate and placed onto a new 12-well plate. Immediately after that, the apical medium was collected using a 1-ml syringe with a short needle (305122, BD Biosciences) and then immediately injected into a 20-ml pre-reduced and autoclaved HDSP vial (C4020-201, Thermo Scientific) sealed with 20-mm Crimp Cap (95025-01-1 S, MicroSolv). All the vials were transferred into an anaerobic chamber, where 10 µl of the apical medium was used for CFU counting on agar plates. The rest of the medium was transferred into a 1.5-ml polypropylene tube, where bacterial cells were pelleted in a microcentrifuge (14,000 *g* × 5 min). The supernatant was transferred into a new 1.5-ml tube. All samples were stored at −80 °C until further analysis.

The Transwells were washed twice with PBS^+/+^ (14040182, Thermo Scientific) in both apical and basolateral sides to completely remove the cell culture medium and the residual bacterial cells before brightfield imaging and TEER measurement. After aspirating the PBS^+/+^, 350 μl of 1% 2-mercaptoethanol solution was added to the apical side, followed by incubation for 10 min at room temperature. The solution was mixed with cells homogeneously, and the mixture was collected and stored at −80 °C until further analysis.

### Co-culture of synthetic microbial communities in GuMI

For synthetic communities, all bacterial species (Supplementary Table 2) were grown under anaerobic conditions, as described above. On the day of co-culturing, actively growing bacteria were mixed with equal volume, and the mixture was then diluted 1000 times using 3% YCFA medium in PBS^+/+^ to generate inoculum. Approximately 1 ml inoculum was then injected into the apical channel of the GuMI, which was primed for 24 hours in the same manner as described in the section “Bacteria co-culture with colonic epithelial monolayers”. To avoid bacterial overgrowth, a 3% YCFA medium was used for the co-culture of synthetic communities in GuMI, whereas a 10% YCFA medium was used for the co-culture of single species in GuMI. After the experiment, the whole device was transferred to a biosafety cabinet, and the basal plate was carefully disassembled. The sealed Transwells were individually removed from the apical plate and placed onto a new 12-well plate. Immediately after that, the same procedures in the section “Bacteria co-culture with colonic epithelial monolayers” were followed to determine bacterial density and TEER. Bacterial cell pellet was used to determine the bacterial composition of the synthetic consortia using the previously reported method^72^.

### Multiplex cytokine/chemokine assays

The concentration of autocrine factors, cytokines, and chemokines in the apical media was measured using customized MULTIPLEX MAP assays, 47-plex human cytokine/TH17 panel (EMD Millipore) adapted from the previous protocol^16^. Briefly, samples were measured at multiple dilutions to ensure the measurements were within the assay’s linear dynamic range. We reconstituted the protein standard in the same media and serially diluted the protein stock to generate a 7-point standard curve. Assays were run on a Bio-Plex 3D Suspension Array System (Bio-Rad Laboratories, Inc.). Data were collected using the xPONENT for FLEXMAP 3D software, version 4.2 (Luminex Corporation, Austin, TX, USA). The concentration of each analyte was determined from a standard curve that was generated by fitting a 5-parameter logistic regression of mean fluorescence on known concentrations of each analyte (Bio-Plex Manager software).

### RNA extraction and reverse transcription-quantitative polymerase chain reaction (RT-qPCR)

Prior to RNA extraction, the cell lysate in 1% 2-mercaptoethanol solution was mixed with one volume of 350 µl of 70% ethanol and pipetted to a homogeneous mixture. Then, total RNA was extracted using a PureLink RNA mini kit (ThermoFisher, 12183020) by following the manufacture protocol, except treating samples with PureLink DNase (ThermoFisher, 12185010) during one of the wash steps to remove DNA.

RT-qPCR was performed to quantify gene expression. Briefly, the mRNA was converted to cDNA using the High-Capacity RNA-to-cDNA Kit (Thermo Fisher Scientific, 4387406). TaqMan Fast Advanced Master Mix (Thermo Fisher Scientific, 4444557) and TaqMan probe were mixed in MicroAmp EnduraPlate Optical 96-well fast clear reaction plate with barcode (Thermo Fisher Scientific, 4483485) according to manufacture protocol. TaqMan probes used in this study are available in Supplementary Table 4.

### Immunofluorescence staining

The immunofluorescent staining of the monolayers was carried out based on the procedures described previously^17^. Briefly, monolayers taken off the platform were immediately fixed with 4% formaldehyde for 10 mins following a very gentle sampling of the apical medium. The samples were then permeabilized with 0.2% Triton-X for 10 minutes. After permeabilization, the wells were washed once in PBS^+/+^ and immediately stained overnight with Phalloidin-iFluor 488 Reagent (1:1000, ab176753-300TEST, Abcam) and DAPI (1:1000, 62248, Thermo Scientific) in Blockaid at 4 °C. After washing the samples with PBS^+/+^ for two times, the monolayers were excised and mounted on a coverslip using ProLong Gold antifade reagent (Thermo Fisher, P36930). Mounted samples were imaged with a Zeiss LSM800 confocal microscope.

### TEER measurement

EndOhm-12 chamber with an EVOM2 meter (World Precision Instruments) was used to measure the TEER values.

### Reporting summary

Further information on research design is available in the Nature Research Reporting Summary linked to this article.

### Supplementary information

Supplementary material

Reporting summary

## Data Availability

The data that support the findings of this study are available in supplementary files or from the corresponding authors upon request.
