# Supplementary material for: An immune-competent human gut microphysiological system enables inflammation-modulation by Faecalibacterium prausnitzii
Source: NPJ Biofilms Microbiomes. 2024 Mar 29;10:31. doi: 10.1038/s41522-024-00501-z (PMC10980819; doi:10.1038/s41522-024-00501-z)
Supplement: Supplementary file 1 — Supplementary material [file 41522_2024_501_MOESM1_ESM.pdf]

An immune-competent human gut microphysiological system enables inflammation-modulation by *Faecalibacterium prausnitzii*

Jianbo Zhang,[1,2,3] * Yu-Ja Huang,[1] Martin Trapecar,[1] Charles Wright,[1] Kirsten Schneider,[1] John Kemmitt,[1] Victor Hernandez-Gordillo,[1] Jun Young Yoon,[1,4] Mathilde Poyet,[1,9] Eric J. Alm,[1] David T. Breault,[5] David Trumper,[6] Linda G. Griffith[1,7,8] *

[1] Department of Biological Engineering, Massachusetts Institute of Technology, Cambridge, MA, USA

[2] Swammerdam Institute for Life Sciences, University of Amsterdam, Amsterdam, The Netherlands

[3] Tytgat Institute for Liver and Intestinal Research, Amsterdam Gastroenterology, Endocrinology and Metabolism, Amsterdam UMC, Location Academic Medical Center, Amsterdam, the Netherlands

[4] Department of Mechanical Engineering, Yonsei University, Seoul, South Korea

[5] Department of Pediatrics, Harvard Medical School, Boston, MA, USA

[6] Department of Mechanical Engineering, Massachusetts Institute of Technology, Cambridge, MA, USA

[7] Center for Gynepathology Research, Massachusetts Institute of Technology, Cambridge, MA, USA

[9] current address: Institute of Experimental Medicine, University of Kiel, Kiel, Germany

* correspondence should be addressed to JZ (j.zhang6@uva.nl) ORCID: 0000-0003-3526-4586 and LGG (griff@mit.edu) ORCID: 0000-0002-1801-5548.

Supplementary Note

We inoculated three synthetic communities of 2, 4, and 7 bacterial species (Supplementary Figure 1). After 24 hours of coculture with synthetic communities in GuMI, the monolayers were still intact, with the TEER values above 500 Ω cm^2 (Supplementary Figure 1a), confirmed by microscopic inspection of the monolayers (Supplementary Figure 1b). Despite using a more diluted YCFA medium, the bacterial density of the synthetic community is surprisingly higher than 10^{10} CFU per mL for all synthetic communities (Supplementary Figure 1c). Compared to the starting composition, the bacterial composition shifted from *F. prausnitzii*-dominant to *Bacteroides*-dominant (Supplementary Figure 1d). These results demonstrated the feasibility of GuMI in coculturing the synthetic communities with colonic epithelium while maintaining a high density of bacterial cells. However, optimization, such as adding species from other phyla, is required to create more balanced synthetic communities that better represent native human gut microbiota.[1]

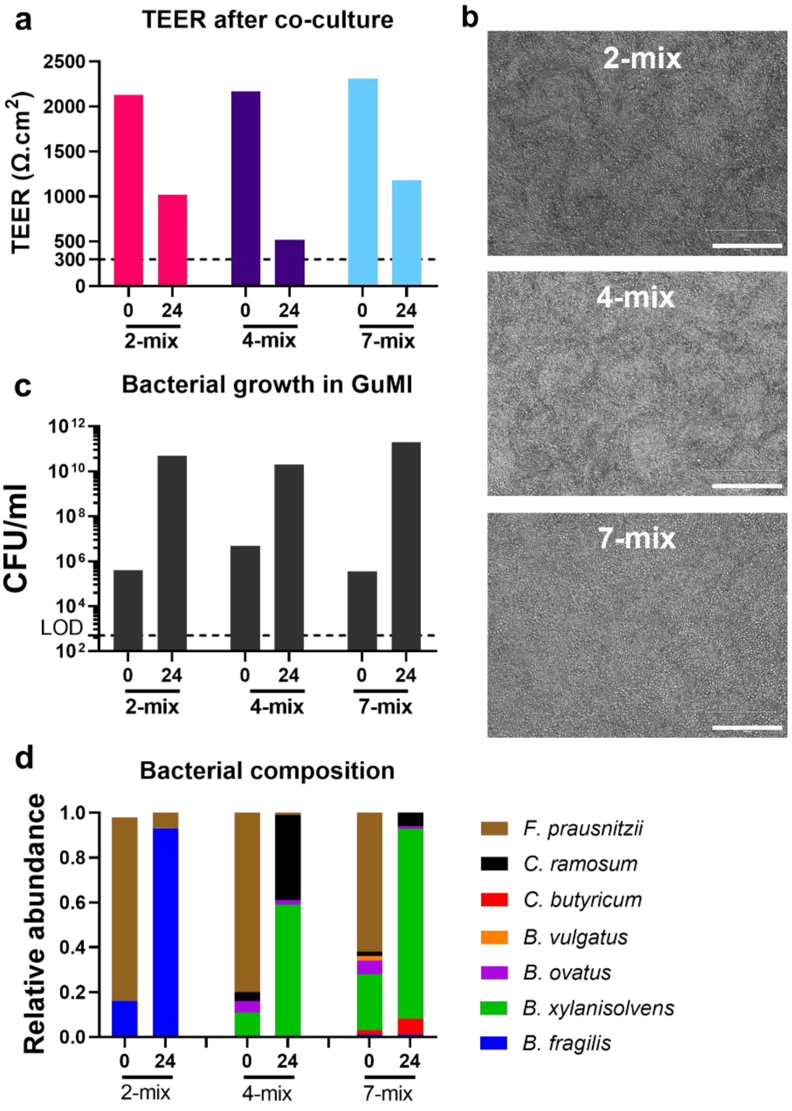

Supplementary Figure 1. Synthetic communities coculture with colonic epithelium in GuMI without immune cells. (a) The TEER values of colonic epithelial monolayer before and after coculture with respective synthetic communities. 2-mix: *F. prausnitzii* and *B. fragilis*. 4-mix: *F. prausnitzii, C. ramosum, B. ovatus,* and *B. xylanisolvens*. 7-mix: *F. prausnitzii, C. ramosum, C. butyricum, B. vulgatus, B. ovatus, B. xylanisolvens,* and *B. fragilis*. The dashed line indicates the threshold of an intact monolayer. (b) Bright-field microscopic images of colonic monolayers after 24 hours of coculture with respective synthetic communities. Scale bar: 300 μm. Bacterial density (c) and bacterial composition (d) in the inoculum (t = 0 h) and after 24 hours of coculture (t = 24 h) in GuMI. LOD in (c) limit of detection.

NNNNNNNNNNNNNNNTTCGGCGGCGTCCTCCTTGCGGTTAGACTACCGACTTCGGGTCCCCCC
GGCTCTCATGGTGTGACGGGCGGTGTGTACAAGGCCCGGGAACGTATTCACCGTGGCATGCTGATC
CACGATTACTAGCAATTCCGACTTCGTGCAGGCGAGTTGCAGCCTGCAGTCCGAACTGGGACGTTG
TTTCTGAGTTTTGCTCCACCTCGCGGTCTTGCTTCTCTTTGTTTAACGCCATTGTAGTACGTGTGTAG
CCCAAGTCATAAAGGGCATGATGATTTGACGTCATCCCCACCTTCCTCCGTTTTGTCAACGGCAGTC
CTGCCAGAGTCCTCTTGCGTAGTAACTGACAGTAAGGGTTGCGCTCGTTGCGGGACTTAACCCAAC
ATCTCACGACACGAGCTGACGACAACCATGCACCACCTGTCTCTGCGTCCCGAAGGAAAATACTGT
TTCCAGCATCGTCGCAGGATGTCAAGACTTGGTAAGGTTCTTCGCGTTGCGTCGAATTAAACCACAT
ACTCCACTGCTTGTGCGGGCCCCCGTCAATTCCTTTGAGTTTCAACCTTGCGGTCGTACTCCCCAGG
TGGATTACTTATTGTGTTAACTGCGGCACTGAAGGGGTCAATCCTCCAACACCTAGTAATCATCGTTT
ACGGTGTGGACTACCAGGGTATCTAATCCTGTTTGCTACCCACACTTTCGAGCCTCAGCGTCAGTTG
GTGCCCAGTAGGCCGCCTTCGCCACTGGTGTTCCTCCCGATATCTACGCATTCCACCGCTACACCGG
GAATTCCGCCTACCTCTGCACTACTCAAGAAAAACAGTTTTGAAAGCAGTTTATGGGTTGAGCCCAT
AGATTTCACTTCCAACTTGTCTTCCCGCCTGCGCTCCCTTTACACCCAGTAATTCCGGACAACGCTTG
TGACCTACGTTTTACCGCGGCTGCTGGCACGTAGTTAGCCGTCACTTCCTTGTTGAGTACCGTCATTA
TCTTCCTCAACAACAGGAGTTTAC

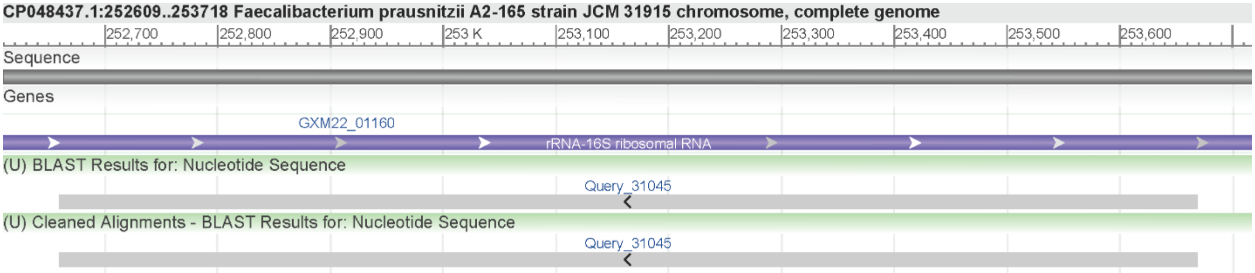

Supplementary Figure 2. The sequence of 5’-3’ of PCR products derived from bacterial DNA in GuMI-APC-FP using Sanger sequencing and the primer pair F8 and 1492R. The identity of *F. prausnitzii* was confirmed using NCBI Blastn with the sequence highlighted in blue as the query sequence (Query_21045).

Supplementary Table 1. The level of cytokines in the apical compartment in the absence of immune cells.

	Conditions						
	apical blank	exp-0412	exp-0509	exp-0412	exp-0412	exp-0509	exp-0509
Analytes	apical blank	GuMI-NB	GuMI-NB	GuMI-FP	GuMI-FP	GuMI-FP	GuMI-FP
EGF	11.39	11.50	OOR <	6.05	4.88	OOR <	1.71
FGF2	OOR <	OOR <	OOR <	OOR <	OOR <	OOR <	OOR <
Fracktalkine	OOR <	OOR <	OOR <	OOR <	OOR <	OOR <	OOR <
G-CSF	8.71	20.60	OOR <	63.71	65.01	OOR <	OOR <
GM-CSF	OOR <	OOR <	OOR <	OOR <	OOR <	OOR <	OOR <
GRO	OOR <	OOR <	OOR <	OOR <	OOR <	OOR <	OOR <
IFNa2	OOR <	2.07	OOR <	OOR <	OOR <	OOR <	OOR <
IFNg	OOR <	OOR <	OOR <	OOR <	OOR <	OOR <	OOR <
IL-10	OOR <	0.27	OOR <	OOR <	0.59	OOR <	OOR <
IL-13	OOR <	OOR <	OOR <	OOR <	OOR <	OOR <	OOR <
IL-15	OOR <	OOR <	OOR <	OOR <	OOR <	OOR <	OOR <
IL-18	OOR <	OOR <	OOR <	0.25	0.28	OOR <	OOR <
IL-1RA	8.69	12.86	4.65	8.09	3.33	7.32	5.31
IL-1a	OOR <	OOR <	OOR <	OOR <	2.50	OOR <	OOR <
IL-21	0.18	0.60	OOR <	2.60	0.24	OOR <	OOR <
IL-8	OOR <	OOR <	OOR <	OOR <	OOR <	OOR <	OOR <
IL12-p40	7.75	7.15	3.89	5.01	5.00	3.68	3.04
IP10	OOR <	OOR <	OOR <	OOR <	OOR <	OOR <	OOR <
MCP-1	OOR <	OOR <	OOR <	OOR <	OOR <	OOR <	OOR <
MCP-3	OOR <	1.75	OOR <	OOR <	OOR <	OOR <	OOR <
MDC	9.00	OOR <	OOR <	12.92	7.03	OOR <	OOR <
MIP-1a	OOR <	OOR <	OOR <	OOR <	OOR <	OOR <	OOR <
MIP-1b	6.24	OOR <	OOR <	OOR <	OOR <	OOR <	OOR <
PDGF-aa	OOR <	OOR <	OOR <	OOR <	OOR <	OOR <	OOR <
PDGF-ab/bb	OOR <	OOR <	OOR <	OOR <	OOR <	OOR <	OOR <
RANTES	OOR <	2.41	2.13	0.51	1.14	OOR <	OOR <
TGFa	OOR <	4.75	OOR <	OOR <	OOR <	OOR <	OOR <
TNFa	OOR <	OOR <	OOR <	1.72	0.58	OOR <	OOR <
TNFb	OOR <	OOR <	OOR <	OOR <	OOR <	OOR <	OOR <
VEGF	OOR <	OOR <	OOR <	OOR <	OOR <	OOR <	OOR <
sCD40L	OOR <	6.28	7.81	14.90	9.80	OOR <	OOR <
IL-4	21.83	28.54	22.78	11.57	37.57	17.93	20.01
IL-22	27.69	31.41	OOR <	41.80	24.75	OOR <	OOR <
Eotaxin	OOR <	OOR <	OOR <	OOR <	OOR <	OOR <	OOR <
Flt-3L	9.09	9.45	OOR <	OOR <	OOR <	OOR <	OOR <
IL-12p70	0.16	OOR <	OOR <	2.35	OOR <	OOR <	OOR <
IL-17E/IL-25	OOR <	OOR <	OOR <	OOR <	OOR <	OOR <	OOR <

IL-17a	OOR <	OOR <	OOR <	OOR <	0.35	OOR <	OOR <
IL-1b	OOR <	OOR <	OOR <	OOR <	0.51	OOR <	OOR <
IL-2	0.29	OOR <	OOR <	OOR <	0.54	OOR <	OOR <
IL-23	OOR <	137.37	OOR <	66.58	OOR <	OOR <	OOR <
IL-3	0.55	0.79	OOR <	0.23	1.13	OOR <	OOR <
IL-33	OOR <	OOR <	OOR <	OOR <	OOR <	OOR <	OOR <
IL-5	0.44	1.26	OOR <	0.29	0.25	OOR <	OOR <
IL-6	OOR <	OOR <	OOR <	OOR <	OOR <	OOR <	OOR <
IL-7	OOR <	OOR <	OOR <	OOR <	OOR <	OOR <	OOR <
IL-9	OOR <	OOR <	OOR <	OOR <	OOR <	OOR <	OOR <
OOR = Out of Range; OOR< = Out of Range Below							

Supplementary Table 2. Key reagents and materials

Name	Supplier	Catalog number
Advanced DMEM/F12	Gibco	12634-010, 500 mL
DMEM/F12	Sigma-Aldrich	D6421-500ML
RPMI 1640	Gibco	11875-085, 1000 mL
HEPES Buffer	Gibco	15630-080, 100x
Penicillin/Streptomycin	Gibco	15140-148, 100x
Glutamax	Gibco	35050-061, 100x
Fetal Bovine Serum, Certified, Heat Inactivated, US Origin	Gibco	10082147
Dimethyl Sulfoxide	Sigma	D2650-100ML
WRN Conditioned Medium	Breault Lab at BCH. Cell line produced by ATCC	CRL-3276 (cell line)
R-Spondin1 Conditioned Media	Breault Lab at BCH. Cell line produced by Sigma-Aldrich	SCC111 (cell line)
B-27 Supplement	Gibco	17504-001, 50x
N-2 Supplement	Gibco	17502-001, 100x
Nicotinamide	Sigma-Aldrich	N0636
N-acetyl L-cysteine	Sigma-Aldrich	A9165
Y-27632 dihydrochloride	BioGems	1293823
SB202190	BioGems; Tocris	1523072; 1264
A83-01	BioGems	9094360
Murine EGF	Peprotech	AF-315-09
Human [leu^{15}]-Gastrin I	Sigma-Aldrich	G9145
Prostaglandin E2	BioGems	3632462
Thiazovivin	Sigma-Aldrich	SML1045
Human Noggin	Peprotech	120-10C
PBS, pH 7.4	Gibco	10010023
Collagen I Rat Protein, Tail	Gibco	A10483-01
10× Trypsin Solution	Sigma	T4549
TypLE Express	Gibco	1260413

UltraPure 0.5M EDTA, pH 8.0	Invitrogen	15575020
Growth Factor-Reduced, Phenol Red Free Matrigel	Corning	356231
Cell Recovery Solution	Corning	354253
24-Well Cell Culture Plates	Olympus Plastics	25-107
Fresh Whole Blood with CPDA-1 Anticoagulant	Research Blood Components LLC	N/A
SepMate PBMC Isolation Tubes	Stemcell Technologies	85450
EasySep Human Monocyte Enrichment Kit without CD16 depletion	Stemcell Technologies	19058
EasySep Human Naïve $CD4^{+}$ T Cell Isolation Kit II	Stemcell Technologies	17555
Recombinant Human IL-4	BioLegend	574004
Recombinant Human GM-CSF/CSF2 (Carrier Free)	BioLegend	572903
Recombinant Human M-CSF/CSF1 (Carrier Free)	BioLegend	574804
Retinoic Acid	Sigma	R2625-50MG
Trypan Blue	Invitrogen	T10282
Countess II Automated Cell Counter	Invitrogen	AMQAX1000
Blockaid	Thermo Scientific	B10710
**Bacterial species**		
Bacteroides vulgatus	ATCC	ATCC8482
Bacteroides xylanisolvens	BIO-ML [1]	bq_0049_0032_a5
Bacteroides fragilis	BIO-ML [1]	am_0171_0068_a2
Bacteroides ovatus	ATCC	ATCC 8483
Clostridium butyricum	BIO-ML [1]	bj_0095_0031_f8
Clostridium ramosum	ATCC	DSM 1402
Faecalibacterium prausnitzii	Harvard Digestive Disease Center	DSM 17677

Supplementary Table 3. Composition of the media used in this study.

**Media Name**	**Composition**
Base Medium	Advanced DMEM/F12, 2mM Glutamax, 10mM HEPES, 1x Penicillin/Streptomycin
Antibiotic-free Base Medium	Advanced DMEM/F12, 2mM Glutamax, 10mM HEPES
Organoid Freezing Medium	70% base medium, 20% heat-inactivated FBS, 10% DMSO
Organoid Growth Medium	65% L-WRN conditioned medium, 32% base medium, 1x B-27, 1x N-2, 10 mM Nicotinamide, 500 µM N-acetyl L-cysteine, 10 µM Y-27632 dihydrochloride, 10 µM SB202190, 500 nM A83-01, 50 ng/mL murine EGF, 10 nM human [Leu^{15}]-Gastrin I, 5 nM prostaglandin E2
Washing Medium	DMEM/Nutrient Mixture F-12 Ham, 10% heat-inactivated FBS, 2mM glutamax, 1x Penicillin/Streptomycin
Colon Seeding Medium	65% L-WRN conditioned medium, 32% base medium, 1x B-27, 1x N-2, 500 µM N-acetyl L-cysteine, 10 µM SB202190, 500 nM A83-01, 2.5 µM thiazovivin, 50 ng/mL murine EGF, 10 nM human [Leu^{15}]-Gastrin I, 5 nM prostaglandin E2
Colon Differentiation Medium	20% R-spondin1 conditioned medium, 80% antibiotic-free base medium, 1x B27, 1x N2, 500 µM N-acetyl-L-cysteine, 500 nM A83-01
Dendritic Cell Differentiation Medium	90% RPMI 1640, 10% heat-inactivated FBS, 100 ng/mL GM-CSF/CSF2, 70 ng/mL recombinant human IL-4, 10 nM retinoic acid
Macrophage Differentiation Medium	90% RPMI 1640, 10% heat-inactivated FBS, 100 ng/mL M-CSF/CSF1

Supplementary Table 4. TaqMan probes for the genes tested in this study

Gene symbol	Cat #	Supplier
CCL2	Hs00234140	ThermoFisher Scientific Inc.
CXCL8	Hs00174103	ThermoFisher Scientific Inc.
DEFB1	Hs00608345	ThermoFisher Scientific Inc.
GPR65	Hs00269247	ThermoFisher Scientific Inc.
IDO1	Hs00984148	ThermoFisher Scientific Inc.
INFA1	Hs00855471	ThermoFisher Scientific Inc.
NFKB1	Hs00765730	ThermoFisher Scientific Inc.
RETNLB	Hs00395669	ThermoFisher Scientific Inc.
TLR1	Hs00413978	ThermoFisher Scientific Inc.
TLR2	Hs02621280	ThermoFisher Scientific Inc.
TLR3	Hs01551079	ThermoFisher Scientific Inc.
TLR6	Hs01039989	ThermoFisher Scientific Inc.

Supplementary Reference

1. Poyet, M. *et al.* A library of human gut bacterial isolates paired with longitudinal multiomics data enables mechanistic microbiome research. *Nat. Med.* **25**, 1442–1452 (2019).
